# Twenty-Five Years of Alcohol Epidemiology: Trends, Techniques, and Transitions

**Published:** 1995

**Authors:** Mary C. Dufour

**Affiliations:** Mary C. Dufour, M.D., M.P.H., is deputy director of the Division of Biometry and Epidemiology of NIAAA, Bethesda, Maryland

**Keywords:** AOD consumption, AOD-related (AODR) problems, AOD dependence, epidemiology, research, trend, AODR mortality, government agency

## Abstract

Alcohol epidemiology has developed as a discipline only in the second half of this century. The recognition of alcoholism as a diagnosable disease has permitted extensive studies of trends in alcohol consumption and its consequences throughout the United States.

Epidemiology is the study of a disease, injury, or other health-related condition in human populations and of those factors that increase the likelihood that such a condition will occur (Lilienfeld and Lilienfeld 1980). Thus, the primary aim of alcohol epidemiology is to identify and explain the factors that shape the distribution of alcohol use, abuse, and dependence and the consequences in various populations. Such a goal necessarily involves first defining the distribution of alcohol consumption and its related problems in the population. Describing the nature and magnitude of alcohol’s impact on the individual and on society are necessary for developing effective prevention, intervention, and treatment strategies.

This article examines trends in alcohol consumption and in related factors, such as cirrhosis mortality, in the United States during the past 25 years. It also traces the evolution and maturation of the science of alcohol epidemiology over the corresponding timeframe and speculates on future directions in the field.

## A Historical Perspective

Epidemiology has its roots in the study of infectious diseases. Such research first used the public health model, which involves defining a disease agent, host, and environment, to study and control epidemics. During the early and mid-20th century, medical advances and improvements in sanitation led to the mastery of many infectious diseases, such as tuberculosis. Soon thereafter, cancer and heart disease gained recognition as common killers. This resulted in establishing a new discipline—chronic disease epidemiology. In addition, the field of epidemiology’s focus shifted to assessing the general health of communities under normal conditions rather than only under specific epidemic circumstances.

Alcohol epidemiology as a unique discipline is a relative newcomer to the field, and it marks a logical progression in the evolution of the science of epidemiology. The epidemiology of many infectious diseases is relatively straightforward, having a specific, well-defined, easily identifiable etiological agent (e.g., a bacterium or virus); a clear-cut case identification; a short clinical course; and a quickly effective prevention (vaccination) or treatment (antibiotics). The epidemiology of chronic diseases such as cancer and heart disease is more complex. Alcohol epidemiology, however, is even more intricate and challenging because of the multiplicity of its contributing factors. It encompasses alcohol use, abuse, and dependence as well as countless medical, psychological, social, legal, and economic consequences. Difficulties also arise in determining the incidence of alcohol dependence. To accurately count something, such as the number of alcohol-dependent people in the United States, one must first be able to define it. The large number of definitions in alcohol research adds to the complexity of its epidemiology. And, although no one would argue about whether cancer or heart disease fits the medical model of a disease, some people still would insist that alcohol dependence does not fit this category. Because of the frequent fluctuations in these definitions and concepts, alcohol epidemiology is a new and rapidly evolving discipline.

## Surveillance Epidemiology

Alcohol epidemiology’s complexity is best tackled by dividing the field into disciplines with different foci (see below and sidebar, p. 81). One of the hallmarks of epidemiology is surveillance—that is, tracking a disease, injury, or other health-related condition over time to ensure early detection of epidemics and to measure the impact of prevention and intervention efforts. Important factors tracked in alcohol epidemiological surveillance include apparent per capita alcohol consumption, cirrhosis mortality, alcohol-related traffic deaths, alcohol-related morbidity among patients discharged from short-stay community hospitals (i.e., those, excluding military or Veterans Affairs hospitals, wherein patients remain for less than 30 days), and use of alcoholism treatment services (the limitations of these approaches are discussed below).

**Epidemiology: Four Research Perspectives**

Alcohol epidemiology has developed as a conglomeration of four major epidemiological perspectives: psychosocial epidemiology, psychiatric epidemiology, chronic disease epidemiology, and epidemiological sociology. Research conducted using each perspective seeks slightly different information from study participants. In this manner, the four disciplines complement each other in revealing drinking patterns and problems among the U.S. population.

***Psychosocial Epidemiology***

Historically, psychosocial epidemiology and psychiatric epidemiology (discussed below) share common roots. Prior to World War II, both disciplines relied on key community and medical informants (i.e., professionals in a position to know the numbers of people hospitalized with or suffering from a condition of interest) and agency records for information that defined alcohol-related trends in the population. Following World War II, a second generation of studies evolved that used objectively scored measures of psychopathology, psychiatrists’ evaluations of profiles compiled from written interview responses, and responses of community respondents personally interviewed by psychiatrists. The psychosocial epidemiology perspective espouses the concept that distinct psychiatric disorders, including alcohol-use disorders, are merely different manifestations of common etiological factors, particularly social stress. Consistent with this concept of social causation was the unitary concept of mental illness, in which psychiatric disorder was postulated to fall along a continuum as opposed to being seen as a collection of distinct disorders. This perception clearly differs from the simple cause-and-effect system used in traditional medicine. In keeping with the dimensional approach to the measurement of psychopathology, psychosocial epidemiologists rely on the psychometric tradition of psychology, wherein researchers depend on self-reports from subjects who answer closed questions by selecting from answers given in a multiple-choice format (Grant in press).

***Psychiatric Epidemiology***

In contrast with psychosocial epidemiology, psychiatric epidemiology measures mental disorders, including alcohol use disorders, primarily by categorizing them. By providing a category for alcohol-use disorders, this perspective accepts alcoholism as a medical disease entity. The psychiatric epidemiology perspective is based on the clinical examination tradition (i.e., interviews with patients) typical in psychiatry. Most early semistructured clinical interviews either entirely excluded or poorly represented alcohol-use disorders. By comparison, more recent interviews incorporate questions about alcohol-use disorders.^1^

As psychiatric epidemiology has evolved, two series of interview instruments have emerged—one designed to be administered by clinicians and the other developed for use by lay interviewers. The latter series consists of fully structured clinical interviews that yield computer-generated diagnoses of alcohol-use disorders (Grant in press).

***Chronic Disease Epidemiology***

Traditionally, this perspective has focused on such medical maladies as heart disease and cancer. Data on various chronic illnesses, as opposed to mental disorders, have been gathered since the turn of the century. However, information on alcohol use, symptoms, and consequences was not collected routinely until the early 1970’s, because alcohol dependence was not viewed as a chronic disease. By sponsoring regular surveys, the National Institute on Alcohol Abuse and Alcoholism (NIAAA) has played an important role in establishing alcohol dependence in this category.

***Epidemiological Sociology***

This perspective represents the synthesis of several epidemiological approaches to the study of alcohol use and abuse and their consequences. Here, use and consequences are studied independently rather than being treated as one psychiatric condition. The 1960’s marked the beginning of systematic epidemiological sociological surveys of the general population throughout the United States. Most of these national and community studies were sponsored by NIAAA and its predecessor within the National Institute of Mental Health. Researchers at the Alcohol Research Group in Berkeley, CA, have conducted, at approximately 5-year intervals, eight national surveys as well as numerous community studies since the first one was completed in 1965. These researchers have invested much effort in maintaining some degree of comparability across surveys despite changing definitions and conceptualizations of alcohol-use disorders (Grant in press).

—Mary C. Dufour

1Changes over the past 25 years in the definitions of many psychiatric disorders have resulted in the continual need to develop new instruments to assess evolving criteria. For example, criteria for alcohol-use disorders appearing in the third edition of the *Diagnostic and Statistical Manual of Mental Disorders* (DSM-III) published in 1980 have been modified significantly in the revised edition of DSM–III in 1987 and the DSM–IV in 1994. Likewise, criteria in the ninth revision of the *International Classification of Diseases* published in 1977 have been modified substantially in the tenth revision published in 1992.

ReferenceGrantBFEpidemiologyAnnual Review of Addiction Research Treatmentin press

### Apparent Per Capita Alcohol Consumption

This measure provides a global gauge of societal alcohol consumption and is calculated by dividing the alcoholic beverage sales data from every State and the District of Columbia (or shipment data from major beverage industry sources in cases in which States did not provide sales data) by the U.S. population age 14 and older^1^ (Williams et al. 1994).

**Figure 1:**
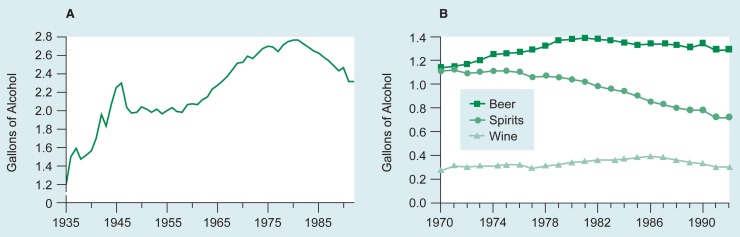
Apparent per capita alcohol consumption statistics calculated from beverage sales data from the 50 States and the District of Columbia. These statistics reveal the average number of gallons of alcohol consumed by a person in the United States. Graph A shows total per capita alcohol consumption in the United States from 1935 to 1992. Graph B shows per capita alcohol consumption by beverage type in the United States from 1970 to 1992. SOURCES: Doernberg and Stinson 1985; Williams et al. 1994.

After Prohibition in the United States, per capita alcohol consumption increased throughout the 1960’s and 1970’s, peaking in 1980 and 1981 at just below 2.8 gallons of pure alcohol per year. During the remainder of the 1980’s, per capita consumption showed a 12-percent decrease, the only sustained decrease since Prohibition (figure 1; Williams et al. 1994). In 1991 per capita consumption experienced one of its largest annual decreases ever, dropping to 2.31 gallons from 2.46 in 1990. In 1992 it remained level at 2.31 gallons, the lowest amount since 1965 (Williams et al. 1994).

### Cirrhosis Mortality

**Figure 2:**
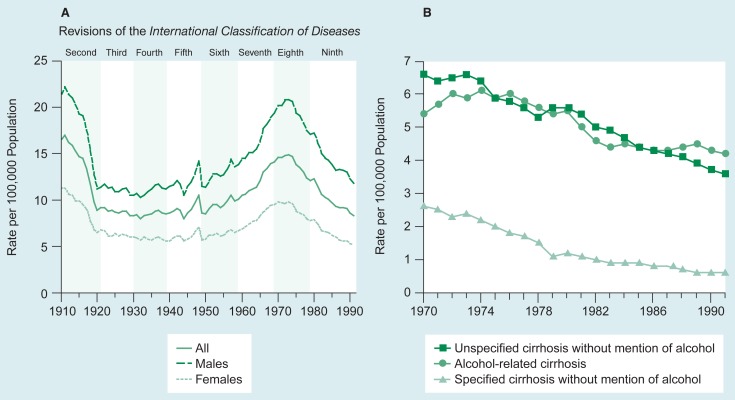
Fluctuations in death rates from liver disease (i.e., cirrhosis) in the United States. Graph A shows the number of men and women who died from cirrhosis from 1910 to 1991. Data were collected from death registrations of States from 1910 to 1932 and of the United States from 1933 to 1991. Graph B shows the number of people who died from cirrhosis from 1970 to 1991. SOURCE: Savage et al. 1994.

Among alcohol-related chronic medical conditions, alcoholic liver disease (i.e., alcoholic cirrhosis) is the leading cause of death. Data regarding deaths from cirrhosis (which includes but is not limited to alcoholic cirrhosis) are compiled using national death records collected annually by the National Center for Health Statistics (NCHS). In 1991, 25,562 people in the United States died from cirrhosis (Savage et al. 1994), making it the 11th leading cause of death (NCHS 1994). Cirrhosis mortality peaked at a 40-year high in 1973 and declined 36.1 percent by 1991, the most recent year for which data are available (figure 2).

### Alcohol-Related Traffic Deaths

**Figure 3:**
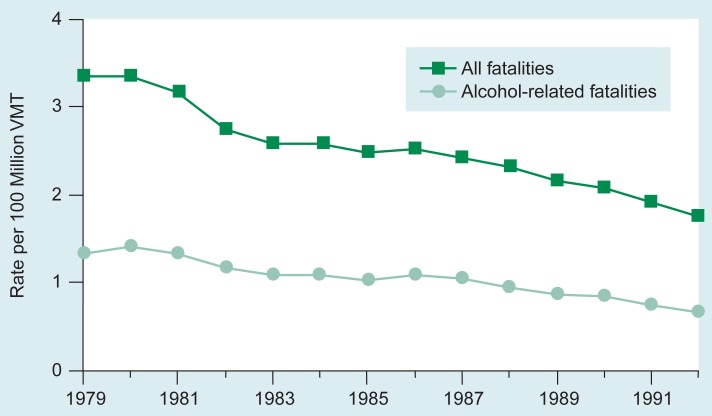
Total and alcohol-related traffic fatality rates per 100 million vehicle miles traveled (VMT) in the United States during 1979 to 1992. SOURCE: Zobeck et al. 1994.

Traffic crashes are the leading cause of death in the United States for people ages 1 through 34 (NCHS 1994). Since 1979 an average of 45,000 people per year have died in traffic crashes, and alcohol involvement in these deaths is estimated to be as high as 45 percent (figure 3; National Highway Traffic Safety Administration 1994). The primary data source for tracking alcohol-related traffic deaths is the U.S. Department of Transportation’s (USDOT’s) Fatal Accident Reporting System (FARS).^2^ At its inception in 1966, one of USDOT’s first activities was a study of the relationship between alcohol and highway safety. Study results (US-DOT 1968) demonstrated that even moderate drinking and driving was associated with greatly increased crash risk and that heavy drinkers apparently played a major role in alcohol-related crashes.

During 1992 the proportion of traffic deaths that were alcohol related reached a 14-year low of 37.4 percent (Zobeck et al. 1994). The number of male drivers involved in alcohol-related fatal crashes decreased 30 percent between 1979 and 1992, whereas the corresponding number of female drivers increased 4 percent over this period (Zobeck et al. 1994).

### Alcohol-Related Morbidity Among Patients Discharged From Short-Stay Community Hospitals

Alcohol-related hospital discharge data are tracked with data obtained from the National Hospital Discharge Survey series, a national sample of hospital discharge episodes, conducted on an ongoing basis since 1965 by NCHS. Current surveillance of alcohol-related discharges began in 1979, the year in which the *International Classification of Diseases, Ninth Revision, Clinical Modification* (ICD–9–CM) was implemented for coding hospital records in the United States. In 1992 (the most recent year for which data are available) there were a total of approximately 28,420,000 discharges for persons age 15 and older from short-stay hospitals, with approximately 423,000 (1.5 percent) of these discharges having an alcohol-related diagnosis that was listed first in the records and approximately 1,175,000 (4.1 percent) having an alcohol-related diagnosis listed anywhere in the records (for each discharge episode, up to 7 diagnoses may be listed). Alcohol-dependence syndrome composed the majority (64 percent) of first-listed diagnoses, followed in order by cirrhosis (17 percent), alcoholic psychoses (12 percent), and nondependent alcohol abuse.

The order of these first-listed alcohol-related diagnoses has remained relatively constant over the 14 years from 1979 through 1992. Overall, hospital discharges with alcohol-related diagnoses demonstrated little significant change over this period (Caces et al. 1994).

### Use of Alcoholism Treatment Services

The past 25 years have witnessed dramatic increases in the number of people using alcoholism treatment services. Statistics from the National Drug and Alcohol Treatment Utilization Survey reveal that over the interval from 1979 to 1991, the number of people in treatment on a given day during the year almost doubled from approximately 293,000 people in treatment on April 30, 1979 (National Institute on Alcohol Abuse and Alcoholism [NIAAA] 1980), to approximately 575,000 on September 30, 1991 (National Institute on Drug Abuse 1992). Membership in Alcoholics Anonymous increased in an equally dramatic manner, rising from approximately 445,000 members in 1979 to nearly 980,000 in 1989 (General Services Board of Alcoholics Anonymous 1991).

### Beyond Surveillance

Data used for surveillance must be easy to quantify, cost-effective to obtain, and available on a periodic (preferably annual) basis. Factors mentioned above, such as cirrhosis, meet these criteria and, in doing so, provide much useful information on alcohol use and alcohol-related consequences. Indeed, rates of all cirrhosis mortality have correlated very well with levels of alcohol consumption in many time periods and many nations. In the United States, death rates from cirrhosis decreased nearly 50 percent within 4 years of Prohibition enactment in 1916, remained low during Prohibition, and began to increase steadily after its repeal in 1932 (Dufour et al. 1993).

#### Limitations of Surveillance Data

Surveillance measures, including estimated per capita alcohol consumption, are quite crude. For example, not all alcoholic beverages produced are actually consumed, and illicit alcohol production, home production, and tax-free personal imports usually are not counted. Most importantly, although per capita consumption fairly accurately reflects the total alcohol volume consumed in a particular geographical area, it provides no information about how consumption is distributed among people in that area; instead, these data assume that all people consume equal amounts.

In reality, not all people drink alcohol, and in fact, the small segment of the population that drinks most heavily has the greatest impact on the total volume of consumption. One person who consumes 10 drinks per day will contribute as much to the total volume as 70 people who consume a single drink each once per week. Clearly, different types of epidemiological studies must be conducted to examine these individual differences.

Likewise, when examining cirrhosis mortality data, alcohol researchers seek to ascertain the number of cirrhosis deaths attributable to alcohol. Because not all cirrhosis is alcohol related, considering all cirrhosis statistics as representative of alcoholic cirrhosis overestimates alcohol’s impact. However, presenting only cirrhosis statistics specifically identified as alcohol related seriously underestimates the true magnitude of the problem. The “alcohol relatedness” of most conditions is grossly underestimated on death certificates for a variety of reasons, including the certifying physician’s lack of familiarity with the decedent’s drinking history and the physician’s desire to protect survivors from the stigma of being identified as the family of an alcoholic (Dufour et al. 1993). The actual number of alcoholic cirrhosis deaths lies somewhere between these two measures, and other data sets, combined with national death records, are required to arrive at this number.

## A Multiplicity of Research Traditions

When discussing trends in national drinking patterns and problems over the past 25 years, it is useful to examine the contexts, volumes, and patterns of drinking that lead to specific alcohol-related problems. Seeking these kinds of information leads from surveillance epidemiology, which defines the prevalence of alcohol consumption and related problems in the population, to survey research. When preparing to conduct a study of drinking contexts and factors, researchers’ conceptualizations of alcohol-related problems will determine how drinking-related questions on surveys are framed and what actual data will be obtained.

### Population-Based Survey Research

A critical advantage of survey data over consumption statistics (such as those in figure 1) is that each respondent’s drinking patterns are recorded separately and can be correlated with other personal characteristics. Modern alcohol epidemiology is a conglomeration of distinct epidemiological disciplines, each of which attempts to describe the problem of alcoholism in the United States from a different etiological perspective (see sidebar, p. 81). Thus, surveys differ with respect to their goals and findings depending on which epidemiological perspectives they are based.

#### Looking Back at Survey Research

Rigorous modern survey research methodology first was applied to an in-depth study of drinking practices in a survey of college students conducted in 1949 (Straus and Bacon 1953). This study was remarkable at the time because it included questions about frequency of drinking, quantities typically consumed per occasion, and even the occurrence of some types of problems resulting from drinking. Beginning in the 1960’s with the work of Cahalan and colleagues (1969), nationwide surveys of drinking practices have applied full statistical sampling methods, which allow the results to be projected with a known degree of confidence to the entire adult household population of the country. Since then, periodic surveys conducted by the NIAAA-sponsored alcohol research center in Berkeley, CA, not only have made it possible to keep up to date on American drinking practices but also to examine changes in these practices over time.

The results of these studies indicate either stability in drinking levels or a slight increase in the prevalence of heavier drinking among men over the period from 1964 to 1984 (Clark and Hilton 1991). Looking at more recent trends, Williams and DeBakey (1992) examined changes in alcohol consumption in the United States from 1983 to 1988 using the 1983 and 1988 National Health Interview Surveys (NHIS; conducted by the NCHS) and found an increase in abstention and a decrease in heavier drinking.

The large number of respondents in both of these surveys (the 1983 NHIS included 22,418 respondents and the 1988 NHIS included 43,809 respondents) made it possible to explore trends separately by gender as well as other sociodemographic characteristics often associated with variations in alcohol consumption. For women, decreases in heavier drinking were found among those 18 to 44 years old, those employed, and those divorced or separated or never married. For men, decreases were found among those employed, those with a family income of $25,000 or more, and those married or divorced or separated (each of these factors contributed significantly to decreasing heavier drinking on its own).

#### NIAAA’s Role

Traditionally, questions about alcohol in study surveys have consisted of a limited number of items on drinking practices, problems, and reasons for drinking. However, as NIAAA has played an increasingly active role in designing and sponsoring these surveys, the detail and complexity of the alcohol-related data have improved dramatically, progressing from minimal quantity and frequency questions in the late 1970’s to 120 questions on the Alcohol Supplement of the 1988 NHIS (Grant in press). The 1988 NHIS is especially noteworthy because it represents a step in the maturation of alcohol epidemiology, signaling the recognition of alcoholism as a diagnosable disease (see sidebar, p. 81). The NHIS is a mainstream national health survey instrument from which actual diagnoses of alcohol abuse and dependence based on criteria listed in the American Psychiatric Association’s *Diagnostic and Statistical Manual of Mental Disorders, Third Edition, Revised* (DSM–III–R) could be made in a large national general household population.

NIAAA has played a critical role in numerous other NCHS-directed national surveys. The 1983 Hispanic Health and Nutrition Examination Survey marked the first national survey of subpopulations of Hispanic-Americans in the United States that included alcohol consumption and health consequences. The NIAAA-supported Alcohol Epidemiologic Data System (AEDS; see box, this page) had major responsibility for refining and analyzing the alcohol-related data from this survey. NIAAA also participated in the Epidemiologic Followup Study of the National Health and Nutrition Examination Survey (NHANES—a nationwide survey of medically defined illness ascertained through physical examination), a long-term longitudinal followup of participants in the 1971–74 NHANES I. These individuals were followed up in 1982–84, 1986, 1987, and 1992–93, allowing epidemiologists to examine morbidity and mortality as they related to alcohol consumption over the participants’ lifespans. NIAAA’s participation in the 1986 National Mortality Follow-back Survey, which examined a 1-percent sample of all deaths occurring in the United States in 1986 (using death records, hospital records, and next-of-kin interviews on lifetime alcohol consumption and many other health-related questions), has enabled a detailed examination of the alcohol-relatedness of cirrhosis deaths.

**The Alcohol Epidemiologic Data System**

In 1988 it was estimated that 15.3 million people in the United States over age 18 abused and/or were dependent on alcohol. How many were women? How many attended alcoholism treatment services? Answers to these and other questions about alcohol are available to both the government and the public through the National Institute on Alcohol Abuse and Alcoholism’s (NIAAA’s) Alcohol Epidemiologic Data System (AEDS).

Established in 1977 to acquire and analyze epidemiological data on alcohol-related subjects for use by NIAAA, AEDS has developed an extensive repository of statistics on problem drinking, consumption patterns, mortality, morbidity, and other relevant issues. AEDS operates under the auspices of NIAAA’s Division of Biometry and Epidemiology and assists NIAAA’s alcoholism surveillance effort by providing researchers with current drinking-related data as well as census and vital statistics information.

In addition to publishing research reports based on its analyses, AEDS manages the Quick Facts electronic bulletin board, which enables anyone with a computer and modem to access up-to-date facts on alcohol-related topics, such as abuse and dependency statistics and information on the costs (i.e., marketing expenses) of alcohol.

Quick Facts uses a bulletin board system (BBS) software package and does not require an online fee, although out-of-area users must pay long-distance telephone charges. Quick Facts is available using the following specifications:

BBS number: (202) 289–4112 (in the United States)
Modem settings: 14,400 (or lower) bps, N (no parity), 8 (data bits), 1 (stop bit)

Quick Facts also is available on the Internet through *telnet fedworld.gov* (192.239.93.3) or *www fedworld.gov* for worldwide web users. From FEDWORLD, proceed to HEALTH MALL, then to Health Gateway Systems, and select #118 for Quick Facts.

—Kathryn G. Ingle

### National Longitudinal Alcohol Epidemiologic Survey

The most recent national information on drinking patterns and problems is derived from the 1992 National Longitudinal Alcohol Epidemiologic Survey (NLAES). NLAES is a nationwide household survey with 42,862 respondents designed and sponsored by NIAAA and fielded by the Bureau of the Census. This study is a landmark in alcohol epidemiology for several reasons. The NLAES is the largest survey ever to focus primarily on alcohol and other drug use and abuse disorders and the first of its magnitude to be conducted by NIAAA. In addition to measuring patterns of use, NLAES is designed to assess the presence of symptoms of past year alcohol abuse and dependence across several diagnostic classification schemes—including DSM–III, DSM–III–R, DSM–IV, and ICD–10—through the use of the diagnostic instrument called the Alcohol Use Disorders and Associated Disabilities Interview Schedule (AU-DADIS). Dr. Bridget Grant of NIAAA and Dr. Deborah Hasin, an NIAAA-funded researcher (Grant et al. 1994), designed the AUDADIS especially for NLAES.^3^

Additional data items on the NLAES include family history of alcohol use disorders; physical health and medical consequences of alcohol use; and use of health care services, including alcoholism and other drug abuse treatment services. Preliminary analyses of the 1992 NLAES revealed that 44 percent of American adults (56 percent of men and 34 percent of women) age 18 and older were current drinkers who had consumed at least 12 drinks in the year preceding the interviews. Twenty-two percent were former drinkers, and 34 percent were lifetime abstainers (22 percent of men and 45 percent of women abstained). These figures reflect an 8-percent decrease in the prevalence of current drinking relative to 1988 (Dawson et al. in press). The proportion of current drinkers (i.e., the full spectrum of drinkers as distinguished from heavier drinkers discussed earlier) decreased with age, increased with income and education, was lower in the South than in other regions, and was lower in rural than in urban areas.

The 1-year prevalence of combined alcohol abuse and dependence in the United States for 1992, as assessed using DSM–IV criteria in the NLAES sample, was 7.41 percent, representing 13,760,000 Americans. In addition, the 1-year prevalence of combined alcohol abuse and dependence was much greater for men (11.0 percent) than for women (4.08 percent). Furthermore, prevalence declined with increasing age. The highest rates for both men (22.07 percent) and women (9.84 percent) were noted among those composing the youngest age group (ages 18 to 29) (Grant et al. 1994). The overall prevalence and corresponding population estimates of alcohol abuse and dependence for 1992 are not substantially different from those for 1984 (Williams et al. 1989) or for 1988 (Grant et al. 1991; 1984 figures are based on DSM–III diagnostic criteria and those for 1988 are based on DSM–III–R diagnostic criteria).

The NLAES represents a major integration of epidemiological research traditions as well as maturation of the field of alcohol epidemiology. Additional ongoing projects in epidemiology sponsored by NIAAA include a cooperative study with the National Heart, Lung, and Blood Institute of the interactions of alcohol consumption with borderline hypertension (for further information and other projects, see table 1).

### NIAAA’s Epidemiology Grants

NIAAA’s epidemiology grant program, which extends grants to researchers at universities and other institutions, continues to flourish, having grown from 3 percent of the entire NIAAA grant program in 1987 to approximately 11 percent today. During 1993 a new Alcohol Research Center grant was awarded to the Research Institute on Addictions in Buffalo, NY, which focuses on alcohol-related clinical and medical epidemiology and serves as an important complement to the well-established Alcohol Research Group in Berkeley, CA, with its focus on social epidemiology.

## Future Trends in Epidemiology at NIAAA

Because of NIAAA’s move to the National Institutes of Health in October 1992, NIAAA now stands as a full and respected partner in the scientific research community. Alcohol researchers are making exciting progress. With a new century and a new millennium just a few short years away, what direction will alcohol epidemiology take in the second 25 years of NIAAA history?

It is unlikely that NIAAA will conduct additional large national surveys on its own in the near future. There are, however, many areas that further studies should address:

- Longitudinal studies, which follow participants at intervals over a period of years or decades, should be conducted to alter the focus of epidemiological research from “snapshots” of drinking problems at a single point in time to descriptions of drinking patterns and consequences over a person’s natural life course. These studies must be carefully designed to be narrowly focused and cost-effective.
- New strategies should be developed for surveying special populations in the United States (including Asian-Americans, American Indians, and Alaska Natives) for whom information on alcohol-related factors is lacking.
- Innovative survey methods should be developed that make use of discoveries in basic science. For example, collecting blood samples from survey respondents may prove useful when laboratory tests for biological markers of alcoholism have been developed. Participants’ responses to survey questions could then be correlated with the presence or absence of biological markers in their blood.
- Advances in computer hardware and software in areas such as mathematical modeling should be explored for their applicability to alcohol epidemiological research.
- More creative and prudent use should be made of existing epidemiological data. For example, meta-analyses of multiple studies can increase scientists’ understanding of relationships between many alcohol-related variables (Fillmore et al. 1991).

Finally, epidemiologists must develop better methods for estimating globally the alcohol-related problems in a community, region, or nation. Although community surveys, such as those cited in this article, are valuable data sources, they generally are limited to households. This means that people not in households at the time of the survey—those who are homeless; incarcerated by the criminal justice system; or residing in rooming houses, hospitals, nursing homes, or mental health facilities—do not participate. These groups are likely to have a much higher-than-average prevalence of heavy alcohol use and alcohol-related problems. Therefore, a methodology to estimate the complete scope of alcohol use and problems would be an important contribution to alcohol epidemiology.

Weisner and Schmidt (1994) have pioneered such a global approach using data collected under the auspices of the Alcohol Research Group’s Community Epidemiology Laboratory. The laboratory includes a variety of data collected from a single county in northern California and uses an assortment of methodological approaches to estimate total alcohol problems in the community. Such techniques would be extremely valuable in describing the total range of alcohol problems in the United States.

By incorporating these suggestions for more effective and efficient epidemiological studies, NIAAA intends to continue a strong tradition of research in this field throughout its second 25 years.

## Figures and Tables

**Table 1:** The National Institute on Alcohol Abuse and Alcoholism’s (NIAAA’s) Ongoing Involvement in Epidemiologic Research

Project Name	Sponsoring Agency/Institute	Purpose of Research
NIAAA Hospital Study^1^	NIAAA	To determine prevalence and impact of cost of alcohol abuse and dependence among patients in short-stay hospitals
1993 National Mortality Followback Survey	NIAAA National Center for Health Statistics Other agencies	To determine alcohol involvement in deaths by injury of teens and young adults, among other purposes
Prevention and Treatment of Hypertension Study	NIAAA National Heart, Lung, and Blood Institute Department of Veterans Affairs	To determine whether reduction of alcohol consumption by heavy-drinking adults with high-normal blood pressure and borderline hypertension will reduce participants’ blood pressure and whether reduced alcohol consumption can be maintained for 2 years

1 The complete name of this study is The Prevalence of Alcohol and Other Drug Abuse and Dependence in Short-Term General Hospitals and the Impact of Abuse and Dependence on Hospital Utilization Charges and Costs.

## Footnotes

1 Although 14 is well below the minimum legal age for the purchase of alcoholic beverages in the United States, numerous studies indicate that many 14-year-olds do drink alcoholic beverages.

2 FARS contains data on all crashes within the United States that involve a motor vehicle traveling on a trafficway customarily open to the public and that results in the death of a vehicle occupant or non-motorist within 30 days of the crash. Although collection of FARS data began in 1975, FARS reports dealing with alcohol-related traffic deaths generally begin with 1979 data. Beginning with 1979 data avoids artificial increases in the reporting of alcohol involvement in FARS that occurred with the introduction of new alcohol variables in 1977 and 1978.

3 The DSM–IV and ICD–10 were not finalized at the time this survey was fielded. However, because of their critical roles on the committees formulating the ICD–10 and DSM–IV, Drs. Grant and Hasin were able to incorporate all of the specific diagnostic criteria necessary to make diagnoses of alcohol-related disorders under both classification schemes (Grant et al. 1994).
